# Direct balloon trocar technique for closed decompression of large predominantly cystic ovarian tumors in adolescents

**DOI:** 10.1007/s00383-025-06002-0

**Published:** 2025-04-12

**Authors:** Silvia Ceccanti, Francesco Morini, Lucia Manganaro, Gabriele Masselli, Giuseppe Stranieri, Denis A. Cozzi

**Affiliations:** 1 Pediatric Surgery Unit, AOU Policlinico Umberto I Hospital, Sapienza University of Rome, Viale Regina Elena, 324, 00161 Rome, RM Italy; 2 Department of Radiological, Oncological and Pathological Sciences, AOU Policlinico Umberto I Hospital, 00161 Rome, RM Italy; 3 Pediatric Surgery Unit, AO Ciaccio-Pugliese Hospital, 88100 Catanzaro, CZ Italy

**Keywords:** Teratoma, Neoplasms, Ovarian mass, Ovarian preservation, Leak-proof drainage, Children

## Abstract

**Purpose:**

Large ovarian tumors with a predominantly cystic pattern are uncommon in adolescents. This study evaluates the effectiveness of a decompression technique utilizing a balloon trocar to facilitate ovarian-sparing surgery, while preserving oncologic principles, in adolescent patients presenting with such lesions

**Methods:**

A retrospective review of four consecutive cases managed by a single surgeon over a 7-year period ending December 2024. We describe the surgical technique of this innovative approach, and provide patient-specific clinical data including histopathologic findings and follow-up outcomes.

**Results:**

The median patient age was 13 years (range, 10–17), and the median maximum tumor diameter was 17 cm (range, 10–28). The balloon trocar technique enabled a tight seal and closed suction drainage of the tumor cyst fluid, facilitating safe decompression and exteriorization of the mass. All surgeries were completed with negligible morbidity. Histological examination revealed three mature cystic teratomas and one serous cystadenoma. At clinical and ultrasonographic follow-up ranging 8 months to 7 years (median, 6,5 years), all patients remained free of recurrence, maintained regular menstrual cycles with normal-sized preserved ovaries, and expressed high satisfaction with their cosmetic outcomes.

**Conclusion:**

Large predominantly cystic ovarian tumors are generally associated with a favorable prognosis. Preemptive in situ decompression using a balloon trocar represents a simple, effective, and rapid technique to minimize the risk of tumor spillage. This approach facilitates safe ovary-sparing surgery for these large tumors, while also providing excellent cosmetic outcomes.

## Introduction

Ovarian neoplasms are relatively uncommon in children and adolescents, accounting for 1% of all malignant tumors in the pediatric population. There are three main types of ovarian neoplasms: epithelial, germ cell and sex-cord stromal, with the germ cell being the predominant type in the pediatric population. Notably, the vast majority of pediatric ovarian neoplasms are benign in nature. Despite this, many pediatric patients are still managed with oophorectomy, which may be particularly deleterious in terms of fertility preservation, given also that they are at increased risk of developing a contralateral second neoplasm [1]. Therefore, a preoperative risk stratification based on physical examination, imaging studies, and serum tumor markers may effectively help identify lesions that are likely to be benign and appropriate for ovarian-sparing surgery [1]. In this context, large ovarian tumors with a predominantly cystic pattern have shown to be generally benign lesions, which has resulted in a progressive paradigm shift toward ovarian-sparing surgery, yet maintain oncologic principles [2].

Preemptive in situ decompression has proved to significantly facilitate removal of these large tumors through a reasonably small laparotomy. Ehrlich et al. first advocated the rationale for this procedure also in the pediatric population [3], promoting the leak-proof technique initially described in adults [4]. This study evaluates the effectiveness of a decompression technique using a balloon trocar to accomplish ovarian-sparing surgery in adolescents with large ovarian cystic tumors.

## Material and methods

We performed a retrospective analysis of our prospectively maintained database including four consecutive cases with a large predominantly cystic tumor treated by a single surgeon over a 7-year period ending December 2024. Extended follow-up data were optimized via telephone contact and in-person visit. Given the primarily descriptive nature of the study and the small number of participating subjects, statistics was limited to measures of central tendency that were calculated as median and range.

### Surgical technique

**Fig. 1:**
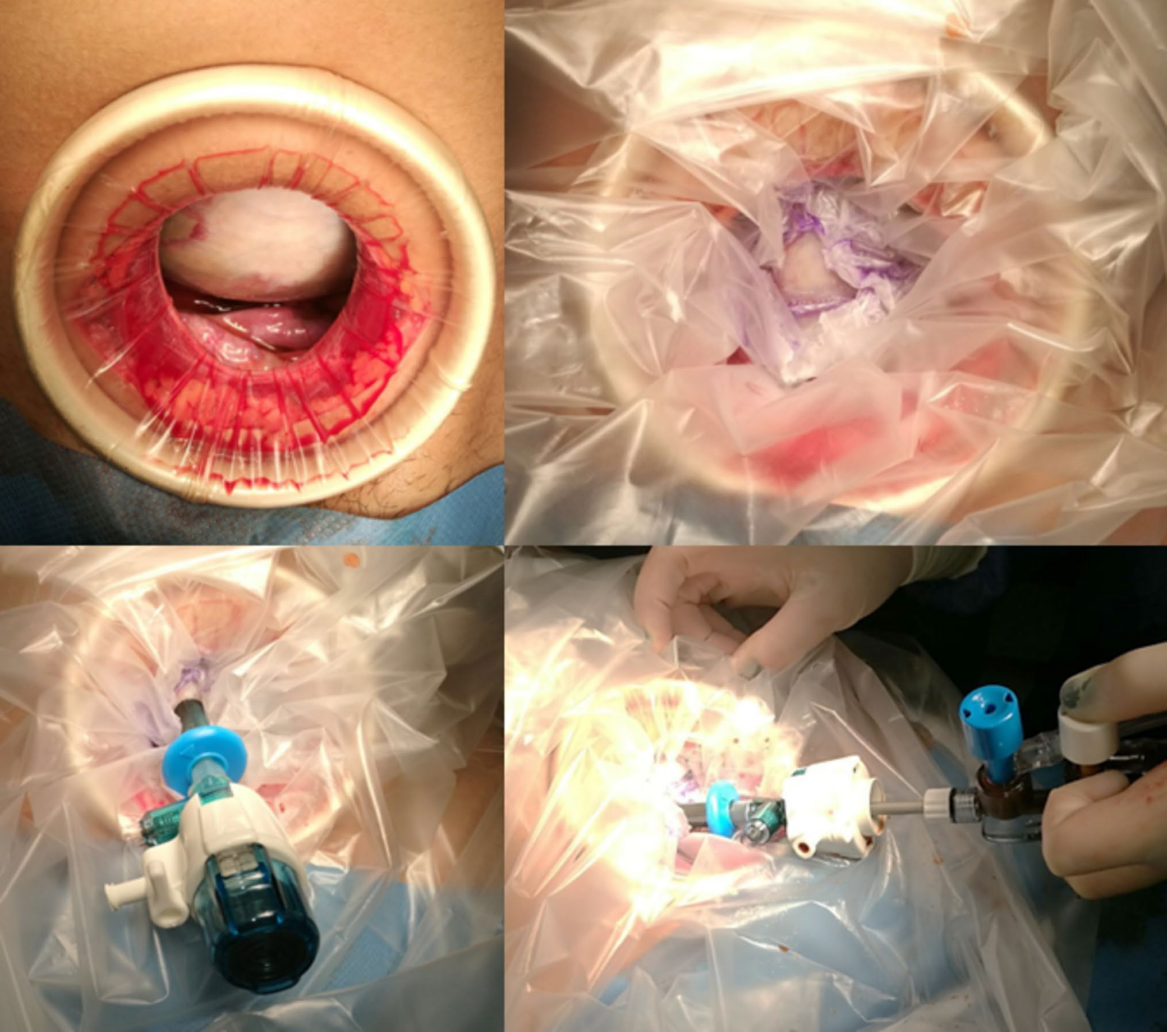
Representative case illustrating the balloon trocar technique for controlled drainage of cystic ovarian tumors. (Top panel) Intraoperative view of the anterior surface of the tumor that is exposed through a small non-muscle-cutting suprapubic laparotomy (left); a sterile plastic drape is attached to the exposed surface of the tumor using cyanoacrylate skin adhesive (right). Note the optimal consolidation of the central portion of the sealed area. (Bottom panel) Intraoperative close-up view of a 5-mm balloon trocar directly inserted into the tumor through the central portion of the sealed area and hold in place by inflating its balloon retention divice (left). Using a suction system for minimally invasive surgery, a controlled drainage of the fluid content of the tumor is obtained in situ, via a substantially closed system that avoids tumor spillage (right)

**Fig. 2:**
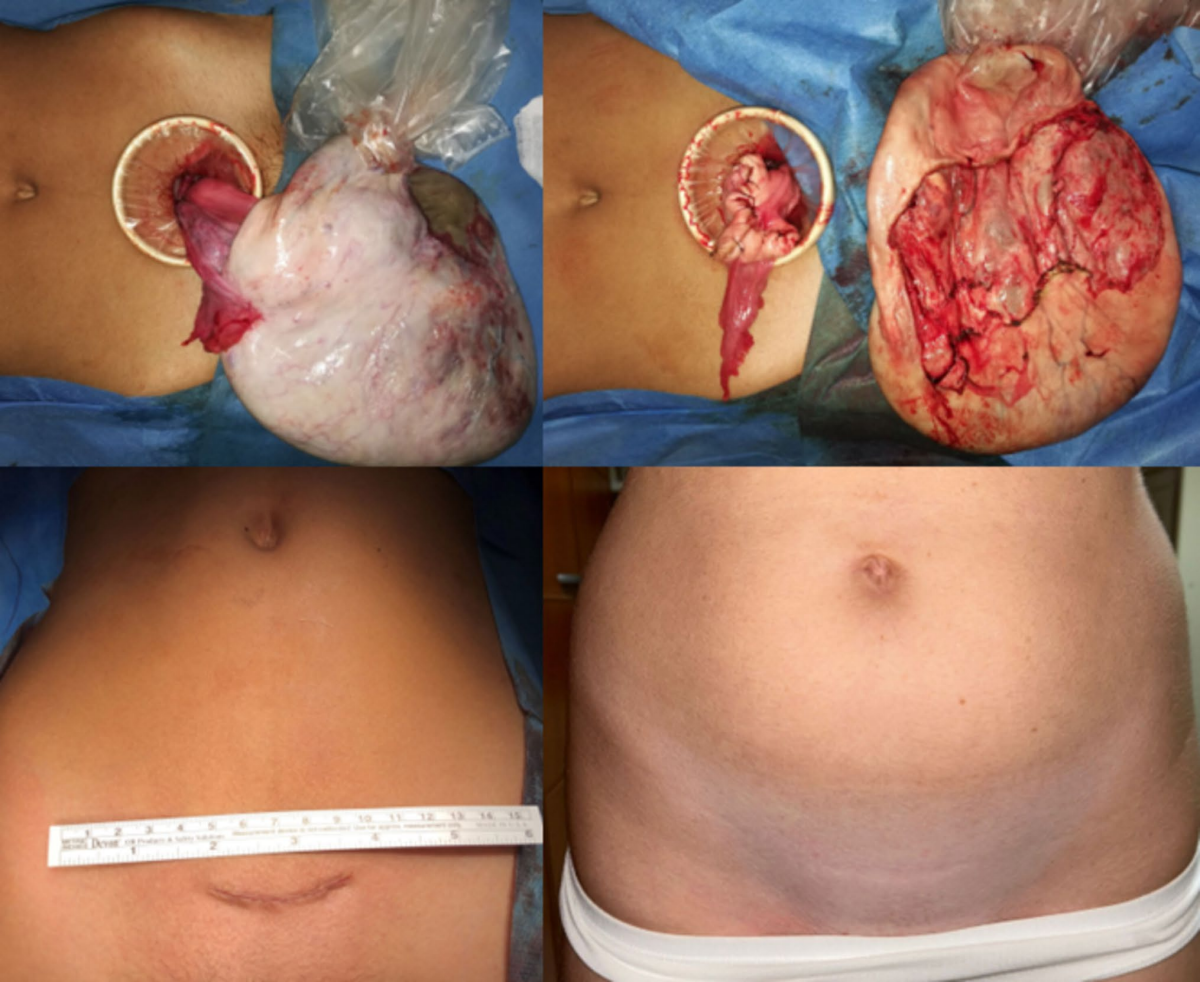
Representative case illustrating ovarian-sparing surgery following drainage of the fluid component of the tumor using the balloon trocar technique. (Top panel) Intraoperative view of the decompressed tumor exteriorized outside the operating field. Note the still firmly attached plastic drape that was initially glued onto the tumor surface for decompression of its fluid content (left). The spared ovarian tissue was approximated to create an oval shape configuration and the tumor was excised without causing rupture (right). (Bottom panel) Close-up view of the surgical scar appearance at the end of surgery (left) and after few years of follow-up (right)

In all patients, we used the following consistent technique, which first entails controlled tumor decompression (Fig. 1). This was achieved through a limited non-muscle-cutting Pfannenstiel skin incision to expose the tumor surface, which we optimized with the insertion of a 360-degree atraumatic wound retractor. After obtaining peritoneal washing, the exposed tumor surface was dried and a sterile plastic drape was glued onto it using cyanoacrylate skin adhesive. A 5-mm balloon trocar was then directly inserted into the tumor through the apex of the consolidated area and hold in place by inflating its stabilizer balloon. Using a suction system for minimally invasive surgery a controlled drainage of the fluid content of the tumor was obtained in situ, until the decompressed tumor could be easily exteriorized outside the operating field. The latter maneuver was facilitated by gentle traction on the trocar. Finally, the tumor was excised by sparing normal ovarian tissue in place, taking appropriate precautions to avoid tumor spillage (Fig. 2). The edges of the spared ovary were approximated and wound closure was carried out in the standard fashion with absorbable material.

## Results

**Fig. 3:**
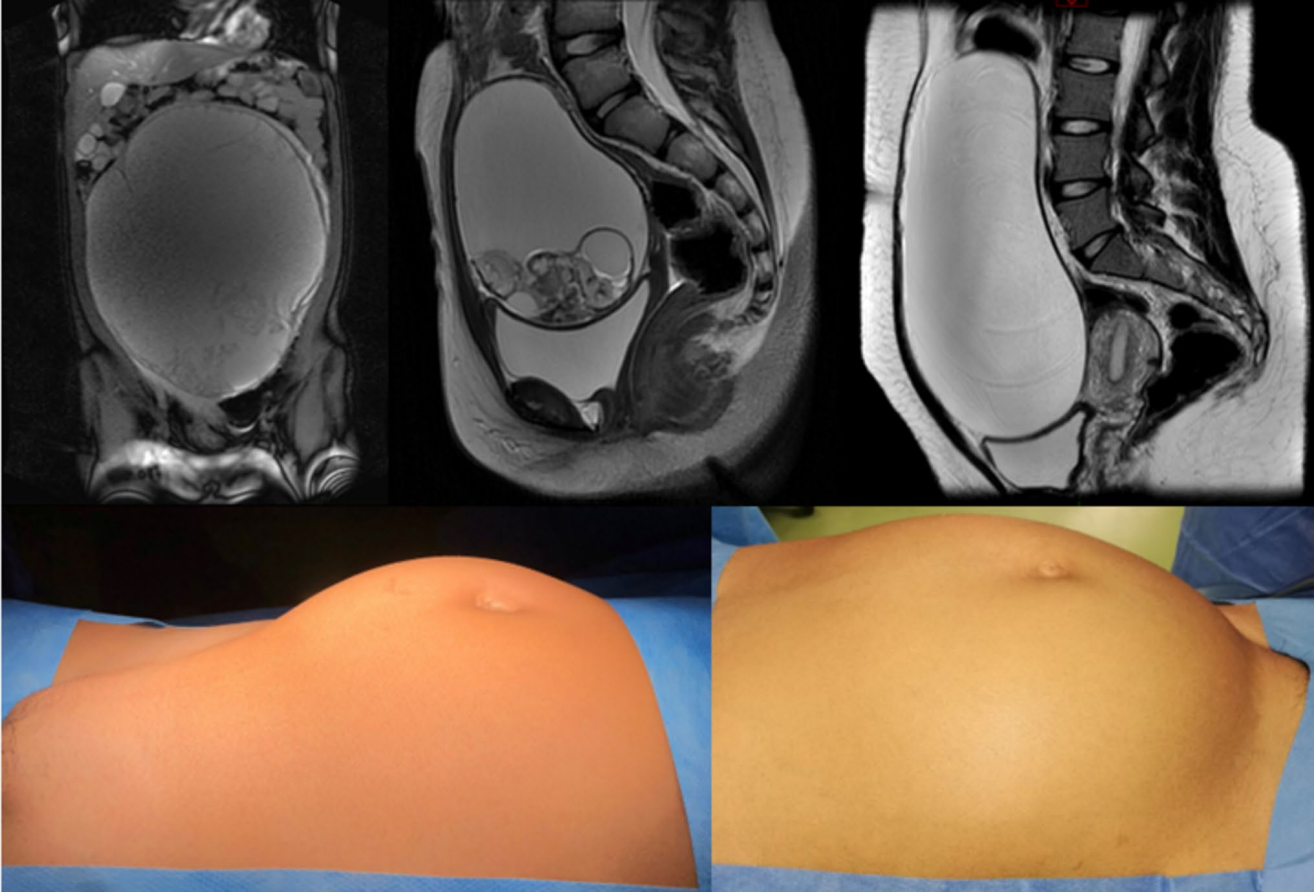
Imaging and physical characteristics of representative cases. (Top panel) MRI appearance of the large ovarian tumor of case #1,2 and 4, respectively. Note the predominantly cystic pattern of the lesions. (Bottom panel) Appearance of the remarkably distended abdomen of the smallest and largest cystic ovarian tumor of present series (case #2 and 1, respectively)

Table 1 summarizes patient characteristics. Two of the four patients had regular periods at diagnosis. Serum beta-HCG, alpha-fetoprotein, CEA, CA19.9, and CA125 levels were normal in all patients. Imaging studies, including abdominal USS and MRI, revealed a predominantly cystic ovarian tumor measuring between 10 and 28 cm in maximum diameter (Fig. 3). All surgeries were carried out without any significant blood loss. At clinical and ultrasonographic follow-up ranging 8 months to 7 years (median, 6,5 years), all patients are free of recurrence, maintain regular periods with normal size of the spared ovaries, and are highly satisfied with their cosmetic result.

**Table 1:** Patient characteristics

Patient	Age (year)	Side	MRI tumor size (cm)	Operative time (min)	Histology	Age at last follow-up (year)
1	15	Rt	28	115	Mature teratoma	22
2	10	Lt	10	69	Mature teratoma	17
3	11	Lt	14	65	Mature teratoma	17
4	17	Rt	20	70	Serous cystadenoma	18

## Discussion

Our small experience is in line with the growing evidence that most of the large ovarian tumors characterized by a predominantly cystic component have a high likelihood of benignity [2]. The most significant predictors for a presumptive benign diagnosis include negative serum tumor markers and the prevailing cystic appearance of the tumor at imaging [1]. Therefore, ovarian-sparing surgery should be encouraged as the preferred option to preserve function of the unaffected parenchyma while not compromising tumor control. Indeed, the major challenge when approaching these lesions resides in their large size that increases the risk of tumor rupture. Nonetheless, a preemptive in situ drainage of the cystic component of the tumor may allow exteriorizing the collapsed tumor outside the abdominal cavity with a relatively low risk of tumor spillage. To achieve this goal, we adopted the leak-proof technique that was originally designed for drainage of large ovarian cystic tumors in adults [4]. The technique entails gluing a sterile plastic film onto the exposed surface of the tumor to reduce the risk of intrabdominal spillage during drainage of the fluid content of the tumor. Notably, Ehrlich et al. first described the leak-proof technique for excision of large cystic ovarian tumors in the pediatric population [3]. However, all the nine treated patients underwent oophorectomy or salpigoophorectomy following controlled drainage of the cystic component of the tumor. Notably, histology disclosed a benign nature of all tumors except one immature teratoma with grade 2–3 immature elements not requiring further treatment. Ovarian-sparing surgery was instead taken increasingly into consideration in subsequent pediatric experiences adopting the same precautionary leak-proof technique [5–8]. In the two largest case series available to date, ovarian-sparing surgery was successfully performed in 5/17 (29%) [5] and 17/23 (74%) [6] of cases, respectively. The increasing adoption of ovarian-sparing surgery is well justified by the excellent oncologic outcomes documented in all but one of the published pediatric cases with large cystic ovarian tumors, regardless of the type of surgery received. The only dismal outcome was reported in a girl with a mucinous cyst adenocarcinoma with evidence of preoperative rupture and positive peritoneal cytology, who died despite receiving adjuvant chemotherapy [6].

We have refined the leak-proof technique by using a balloon trocar, which creates a tight seal and facilitates closed suction drainage, thereby reducing the risk of tumor cell spillage. Previous stratagems for decompressing large cystic ovarian tumors typically involved making a small surgical incision in the center of the glued area and utilizing a standard suction tube for drainage [4, 6]. Unfortunately, this approach can lead to some fluid spillage that accumulates in the protected space covered by the impermeable layer, making effective suctioning necessary for proper management.

Other means of decompression have included the use of a Veress needle [3], a 16-gauge intravenous cannula [5], or a 16-gauge epidural needle [8]. In the latter case, the epidural needle is inserted into the tumor, passing through a nasopharyngeal airway that is glued to the tumor surface to help prevent spillage. However, the use of small-caliber tubes for drainage can be problematic, as they are prone to blockage from the semisolid debris often present in cystic ovarian tumors. Another technique that has been advocated involves the SAND balloon catheter, which contains an inner needle designed to pierce the tumor wall [7]. This allows for the catheter to be advanced and securely positioned inside the tumor by inflating a distal balloon, thereby preventing leakage during drainage. The stability of the catheter is further enhanced when the proximal balloon is inflated, lying just outside the tumor wall. While this approach allows for a closed suction drainage similar to our current method, the SAND balloon catheter is less widely available and more expensive compared to the balloon trocar.

## Conclusion

This study demonstrates that the balloon trocar technique is a safe and effective approach for decompression of large predominantly cystic ovarian tumors while preserving ovarian tissue. The technique entails the use of a trocar having a balloon retention device at its distal portion. Once the trocar is inserted into the tumor, the balloon is inflated and maintained in compression against the internal wall surface of the tumor. This maneuver enhances a tight seal, averts trocar slippage and forms a substantially closed chamber to allow a controlled suction drainage of the tumor content without fluid leakage. This modification is a simple, effective, and rapid technique to minimize tumor spillage and achieve safe ovarian-sparing surgery of these large tumors with excellent cosmetic outcome. Nonetheless, our encouraging preliminary results need further validation in larger prospective studies.

## Data Availability

No datasets were generated or analysed during the current study.

## Abbreviations

Beta-HCG: Beta human chorionic gonadotropin
CA-125: Cancer antigen-125
CEA: Carcinoembryonic antigen
CA 19-9: Cancer antigen 19.9
USS: Ultrasound scan
MRI: Magnetic resonance imaging
